# RNA Polymerase III Regulates Cytosolic RNA:DNA Hybrids and Intracellular MicroRNA Expression*

**DOI:** 10.1074/jbc.M115.636365

**Published:** 2015-01-26

**Authors:** Christine Xing'er Koo, Kouji Kobiyama, Yu J. Shen, Nina LeBert, Shandar Ahmad, Muznah Khatoo, Taiki Aoshi, Stephan Gasser, Ken J. Ishii

**Affiliations:** From the ‡ Immunology Programme and Department of Microbiology, Centre for Life Sciences, and; the § NUS Graduate School for Integrative Sciences and Engineering, National University of Singapore, Singapore 117456,; the ¶ Laboratory of Adjuvant Innovation and; the ** Laboratory of Bioinformatics, National Institute of Biomedical Innovation (NIBIO), Ibaraki, Osaka 567-0085, Japan, and; the ‖ Laboratory of Vaccine Science, World Premier International Immunology Frontier Research Center (iFREC), Osaka University, Suita, Osaka 565-0871, Japan

**Keywords:** Cancer, DNA Damage, Innate Immunity, MicroRNA (miRNA), RNA Polymerase III, RNA Transport, RNA:DNA Hybrid, RNase H, Antibody S9.6

## Abstract

RNA:DNA hybrids form in the nuclei and mitochondria of cells as transcription-induced R-loops or G-quadruplexes, but exist only in the cytosol of virus-infected cells. Little is known about the existence of RNA:DNA hybrids in the cytosol of virus-free cells, in particular cancer or transformed cells. Here, we show that cytosolic RNA:DNA hybrids are present in various human cell lines, including transformed cells. Inhibition of RNA polymerase III (Pol III), but not DNA polymerase, abrogated cytosolic RNA:DNA hybrids. Cytosolic RNA:DNA hybrids bind to several components of the microRNA (miRNA) machinery-related proteins, including AGO2 and DDX17. Furthermore, we identified miRNAs that are specifically regulated by Pol III, providing a potential link between RNA:DNA hybrids and the miRNA machinery. One of the target genes, exportin-1, is shown to regulate cytosolic RNA:DNA hybrids. Taken together, we reveal previously unknown mechanism by which Pol III regulates the presence of cytosolic RNA:DNA hybrids and miRNA biogenesis in various human cells.

## Teaser

**Background:** RNA:DNA hybrids exist in the nucleus and mitochondria but not in the cytosol, except in viral infection.

**Results:** RNA:DNA hybrids exist in the cytosol of various human cells and are mediated by RNA polymerase III (Pol III), which regulates the microRNA machinery.

**Conclusion:** Cytosolic RNA:DNA hybrids are regulated by Pol III.

**Significance:** Previous unknown cytosolic RNA:DNA hybrids may have physiological relevance to miRNA machinery and RNA transport.

## Introduction

RNA:DNA hybrids can occur during transcription and replication of DNA (1). The DNA primase generates short RNA:DNA fragments during replication of the lagging strand (2, 3). Short hybrids also form during the transcription of DNA by RNA polymerases. In contrast, long RNA:DNA hybrids can occur during stalling of the RNA polymerase or during replication of mitochondria DNA (1). Stalling of the RNA polymerases can lead to the formation of R-loops, which consist of long RNA:DNA hybrids and the displaced non-template DNA strand. Long RNA:DNA hybrids also occur in G-quadruplexes, which promote class switch recombination in B-cells (5). Recent evidence suggests that R-loops and G-quadruplexes may occur more frequently than previously assumed and interfere with gene expression and threaten genome stability (6–8). Although many studies have focused on the generation of nuclear RNA:DNA hybrids, it is unclear how nuclear RNA:DNA hybrids are resolved, and their role in diseases related to genomic instability, such as cancer, is not understood.

We recently discovered the presence of ssDNA and dsDNA in the cytosol of B-cell lymphoma cells (9). Inhibition of ATM (ataxia telangiectasia-mutated) and ATR (ataxia telangiectasia- and Rad3-related) kinases, which initiate the DNA damage response (DDR),^3^ leads to the disappearance of cytosolic DNA. Conversely, the levels of cytosolic ssDNA and dsDNA increase in response to DNA damage, suggesting that constitutive nuclear DNA damage and the ensuing DDR induce the presence of cytosolic ssDNA and dsDNA in B-cell lymphoma cells. Cytosolic DNA in B-cell lymphoma cells activates STING-dependent DNA sensor pathways, leading to the expression of ligands for the activating immune receptor NKG2D (natural killer group 2, member D) (9). Delocalized DNA is important for innate immune recognition of pathogens, and recent reports suggest that TLR9 and the NLRP3 inflammasome sense pathogen-derived RNA:DNA hybrids in dendritic cells (10–13). However, whether RNA:DNA hybrids exist in the cytosol of non-infected cells is unknown.

RNA polymerase III (Pol III) is the largest RNA polymerase, consisting of 17 subunits, including a DNA-binding site (14–16). It catalyzes the transcription of genes required for transcription and RNA processing, such as tRNAs, ribosomal 5 S rRNA, and U6 snRNAs. It also transcribes short interspersed elements and repeated elements in the human genome (14). Pol III expression is regulated by oncogene products, tumor suppressors such as p53, and Pol III-associated transcription factors (17–19). Consistent with these observations, Pol III activity is increased in many cancers, including melanomas, myelomas, and carcinomas (20). Although Pol III is present mostly in the nucleus (20, 21), cytosolic Pol III was proposed to play a role in the sensing of AT-rich DNA via the RIG-I (retinoic acid-inducible gene I) pathway (22–24). Despite the regulation of Pol III by genes associated with tumorigenesis, little is known about the role of Pol III in the cellular function of transformed cells.

Here, we identified the presence of cytosolic RNA:DNA hybrids in immortalized and transformed human tumor cells. Chemical inhibition of Pol III abrogated the presence of cytosolic RNA:DNA hybrids in cells. Cytosolic RNA:DNA hybrids were bound by microRNA (miRNA) machinery-associated proteins, such as DDX17 (DEAD (Asp-Glu-Ala-Asp) box polypeptide 17) and AGO2 (argonaute 2). We also identified Pol III-regulated intracellular miRNAs in A549 lung cancer cells. In summary, we demonstrate the constitutive presence of cytosolic RNA:DNA hybrids in a variety of cell lines, and this accumulation depends on Pol III, at least in A549 lung carcinoma cells.

## EXPERIMENTAL PROCEDURES

### 

#### 

##### Cells

The human lung adenocarcinoma (A549), colorectal adenocarcinoma (LoVo and HT29), colorectal carcinoma (HCT116), acute monocytic leukemia (THP-1), human cervix carcinoma (HeLa), and normal lung tissue-derived (MRC-5) cell lines were purchased from American Type Culture Collection (Manassas, VA). Cells were grown in Dulbecco's modified Eagle's medium (Nacalai Tesque) supplemented with 10% fetal bovine serum (Nichirei Biosciences Inc., Tokyo, Japan), 1% penicillin/streptomycin (Nacalai Tesque), and 2% HEPES (Life Technologies). Cells were maintained with 5 μg/ml Plasmocin (InvivoGen) to prevent mycoplasma infection.

##### Reagents and Cell Treatments

Ara-C (cytarabine) was purchased from Wako Chemicals. Aphidicolin, Pol III inhibitor ML-60218, and leptomycin B were purchased from Calbiochem. ATM inhibitor KU60019 (Tocris Bioscience) and ATR inhibitor VE821 (Axon Medchem) were used at 10 μm. PicoGreen dsDNA reagent (Life Technologies) was used at 1:100 dilution. MitoTracker Red CM-H2XRos (Life Technologies) was dissolved in dimethyl sulfoxide (DMSO) and used at 500 nm. Fixed cells were treated with 0.5 units/ml RNase H (New England Biolabs) for 3 h at 37 °C.

##### Immunofluorescence Studies

Cells were fixed in 4% paraformaldehyde (Nacalai Tesque) for 10 min and permeabilized in 0.2% Triton X-100 for 15 min. Nonspecific sites were blocked with 2% goat serum and 1% BSA in 0.2% Triton X-100 for 1 h. Transfected cells were stained with anti-cytochrome *c* oxidase subunit IV (COX IV) antibody (ab16056, Abcam), anti-POLR3G antibody (polymerase (RNA) III (3) (DNA-directed) polypeptide G; LS-C163858, LSBio), or anti-DDX17 antibody (19910-1-AP, Proteintech). The RNA:DNA hybrid-specific antibody S9.6 was a kind gift of Dr. D. Koshland (University of California, Berkeley) (25). The secondary polyclonal antibodies used were Alexa Fluor 488-conjugated goat anti-mouse IgG F(ab′)_2_ fragment (H+L) and Alexa Fluor 555-conjugated goat anti-rabbit IgG F(ab′)_2_ fragment (H+L) (Life Technologies). PicoGreen staining of DNA and MitoTracker staining of mitochondria were performed according to the manufacturer's instructions. Cells were stained with 2 μg/ml Hoechst for 10 min and mounted in mounting medium (Dako). Cell images were taken with a Leica TCS SP2 laser confocal scanning microscope and analyzed using Volocity (version 6.2.1) and Imaris. Micrographs show cells representative of total cell populations.

##### Transfection

A549 cells were transfected with POLR3G siRNA (Qiagen) using Lipofectamine RNAiMAX transfection reagent (Life Technologies) according to the manufacturer's instructions. AllStars negative control siRNA (Qiagen) was used as a control in transfection, and its sequence is proprietary. The POLR3G siRNA sequences used were 5′-AAGGCACACCACTCACTAATA-3′ (siPOLR3G_1) and 5′-TCAGAGTACTCAAGTGTACAA-3′ (siPOLR3G_2).

##### Immunoblotting

Cells were lysed in cold radioimmune precipitation assay buffer (Nacalai Tesque), and lysates were electrophoresed in 4–12% NuPAGE Bis-Tris gel (Life Technologies) and then blotted onto PVDF membranes. Antibodies specific to DDX17 (sc-86409, Santa Cruz), AGO2 (C34C6, Cell Signaling Technology), and GAPDH (M171-3, MBL International) and horseradish peroxidase-conjugated secondary antibodies (Cell Signaling Technology) were used to develop the blots with Immobilon Western chemiluminescent HRP substrate (Millipore). Digital images were acquired using ImageQuant LAS 500 (GE Healthcare).

##### Immunoprecipitation and Mass Spectrometry

A549 cells (2 × 10^6^) were seeded into 100-mm dishes and fixed in 1% paraformaldehyde for 10 min, followed by treatment with 125 mm glycine (Wako Chemicals) for 5 min. Cells were fractionated using a cell fractionation kit (MS861, MitoSciences). The cytosolic fraction was precleared by incubation with 5 μl of protein G-Sepharose beads (GE Healthcare) for 20 min at 4 °C on a rolling shaker. The cleared supernatant was incubated overnight at 4 °C on a rolling shaker with 10 μg/ml RNA:DNA hybrid-specific antibody and 10 μl of protein G-Sepharose beads. Immunoprecipitates were washed sequentially with radioimmune precipitation assay buffer, low salt buffer (20 mm Tris-HCl (pH 8.1), 150 mm NaCl, 0.1% SDS, 1% Triton X-100, and 2 mm EDTA), high salt buffer (20 mm Tris-HCl (pH 8.1), 600 mm NaCl, 0.1% SDS, 1% Triton X-100, and 2 mm EDTA), final wash buffer (20 mm Tris-HCl (pH 8.0), 0.1% SDS, 1% Triton X-100, and 1 mm EDTA), and Tris/EDTA buffer. Beads were resuspended in Tris/EDTA buffer with 1% SDS and incubated overnight at 65 °C to release protein complexes for subsequent gel electrophoresis. For mass spectrometry, similarly processed cell lysates were immunoprecipitated with RNA:DNA hybrid-specific antibody and silver-stained using a Silver Stain Plus kit (Bio-Rad) according to the manufacturer's instructions. Bands of interest were cut out and sent for mass spectrometry analysis at the Osaka University mass spectrometry facility.

##### miRNA Microarray Analysis

A549 cells were treated with 10 μm Pol III inhibitor for 24 h and subsequently treated with 10 μm Ara-C or DMSO for 15 h. DMSO-treated cells served as a control. Total RNA was extracted with TRIzol (Life Technologies) and labeled using a 3D-Gene miRNA labeling kit. The labeled RNA was hybridized to a human miRNA V19 microarray chip containing 2019 miRNA probes and analyzed on a ProScanArray microarray scanner (Toray Industries). miRNA profiles were provided as sample-wise median-normalized data by Toray Industries. Data were further normalized with an all-sample quantile normalization protocol using the corresponding Bioconductor package developed by Bolstad *et al.* (26). Original miRNA profiles consisted of 2019 miRNA probes, of which only a small fraction showed significant expression in any of these experiments. After replacing the missing valued data (no expression observed) with the minimum of all observed expression values, miRNA probes that showed at least 3-fold differential expression between any pair of four experiments were used for further quantitative analysis. Identified miRNA sequences were used to obtain predicted gene targets, as acquired from the public domain resource DIANA-mirPath (27). A *p* value threshold of 0.05 and a MicroT threshold of 0.8 were applied.

##### miRNA Expression Analysis

After RNA extraction, cDNA was synthesized using a miScript II RT kit (Qiagen). miRNA levels were analyzed using assay kits for mature miR-4499 (Qiagen), precursor (includes detection for precursor and primary miR) miR-4499 (Qiagen), and TaqMan primary miR-4499 (Life Technologies) and quantified by quantitative PCR using iTaq Universal SYBR Green Supermix (Bio-Rad) or TaqMan Gene Expression Master Mix (Life Technologies). Precursor miR expression was determined using the equation in Ref. 28.

##### Quantitative Real-time RT-PCR

Experiments were performed as described previously (9). The following primers were used: *XPO1-*5′ (exportin-1), 5′-AGGTTGGAGAAGTGATGCCA-3′; *XPO1*-3′, 5′-GCACCAATCATGTACCCCAC-3′; *KPNB1*-5′ (karyopherin (importin) beta 1), 5′-GACCGACTACCCAGACAGAG-3′; *KPNB1*-3′, 5′-GACTCCTCCTAAGACGACGG-3′; *NUP153*-5′ (nucleoporin 153kDa), 5′-GCCCAAATCTTCCTCTGCAG-3′; *NUP153*-3′, 5′-GAAAGGAGCCACTGAAGCAC-3′; *HPRT1*-5′, 5′-CCCTGGCGTCGTGATTAGTG-3′; and *HPRT1*-3′, 5′-TCGAGCAAGACGTTCAGTCC-3′.

##### Statistical Analysis

For statistical analysis, Student's one-tailed *t* test (*p* < 0.05) was used unless stated otherwise after data were tested positive for normality by the Shapiro-Wilk test. For data that failed the normality test, a non-parametric Mann-Whitney Wilcoxon rank-sum test was used. A *p* value of <0.05 was considered statistically significant.

## RESULTS

### 

#### 

##### Presence of RNA:DNA Hybrids in the Cytosol of Human Cells

**FIGURE 1.**
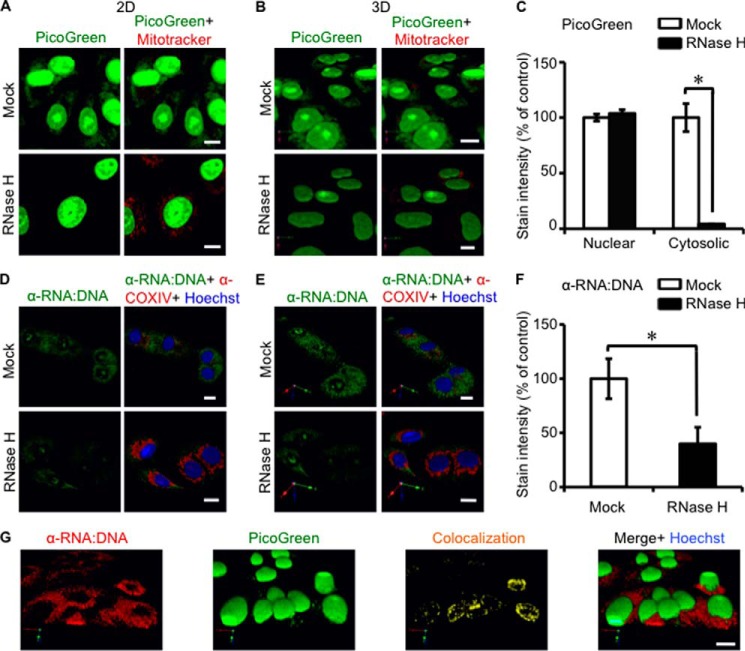
**RNA:DNA hybrids exist in the cytosol of human lung cancer cells.** *A*, the human lung carcinoma cell line A549 was stained with the vital dsDNA-specific dye PicoGreen (*green*) at 10 μl/ml for 1 h and with the mitochondria-specific vital dye MitoTracker (*red*) at 100 nm for 30 min. Samples shown in the *lower panels* were pretreated with 0.5 units/ml RNase H. *Scale bars* = 10 μm. *B* and *C*, three-dimensional isosurface rendering (*B*) and quantification (*C*) of PicoGreen staining in the nucleus and cytosol of the images shown in *A*. A one-tailed Wilcoxon test was performed. *Error bars* represent S.E. *, *p* < 0.05. *D*, A549 cells were stained with the RNA:DNA hybrid-specific antibody S9.6 (*green*) and the mitochondrial marker COX IV (*red*) in the presence of Hoechst (*blue*). Cells shown in the *lower panels* were pretreated with 0.5 units/ml RNase H before staining. *E* and *F*, three-dimensional isosurface rendering (*E*) and quantification (*F*) of RNA:DNA hybrid staining of the images shown in *D. G*, three-dimensional isosurface rendering of staining of A549 cells with PicoGreen (*green*), RNA:DNA hybrid-specific antibodies (*red*), and Hoechst (*blue*). A one-tailed Wilcoxon test was performed.

**FIGURE 2.**
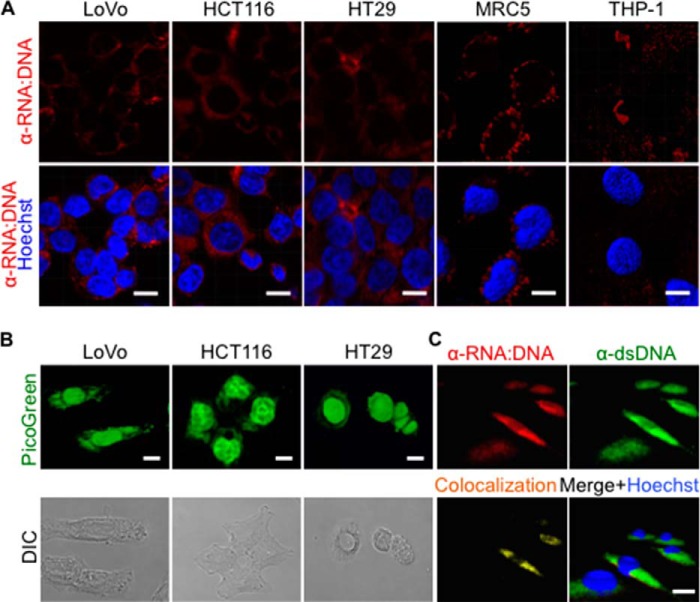
**Presence of cytosolic RNA:DNA hybrids in human tumor cell lines.** *A*, the human colorectal carcinoma cell lines LoVo, HCT116, and HT29; the human acute monocytic leukemia cell line THP-1; the human cervix carcinoma cell line HeLa; and the human normal lung tissue-derived cell line MRC-5 were stained for RNA:DNA hybrids (*red*) in the presence of Hoechst (*blue*). *Scale bars* = 10 μm. *B*, the colorectal adenocarcinoma cell lines LoVo, HCT116, and HT29 were stained with PicoGreen (*upper panels*). Bright-field images (differential interference contrast (*DIC*)) of cells are shown in the *lower panels. C*, three-dimensional isosurface rendering of confocal images of A549 cells stained for the presence of dsDNA (*green*) and RNA:DNA hybrids (*red*) in the presence of Hoechst (*blue*). Co-localization of dsDNA and RNA:DNA hybrids (*yellow*) was determined using Volocity computational image analysis.

We previously reported (9) the presence of cytosolic ssDNA and dsDNA in cancer cells using specific antibodies and the vital dye PicoGreen, which detects dsDNA and RNA:DNA hybrids (29, 30). Here, we sought to test if RNA:DNA hybrids are present in the cytosol of human cancer cell lines. PicoGreen stained DNA in the cytosol of the human lung carcinoma cell line A549 and other cancer cell lines (Fig. 1, *A* and *B*, and Fig. 2, *A* and *B*). Three-dimensional surface rendering of confocal images showed that the majority of extranuclear DNA is present outside of mitochondria (Fig. 1*B*). To analyze if RNA:DNA hybrids contribute to the PicoGreen signals in the cytosol, A549 cells were treated with RNase H, which degrades RNA in RNA:DNA hybrids (31), prior to staining with PicoGreen. Pretreatment of cells with RNase H abrogated the cytosolic PicoGreen signals (Fig. 1, *A–C*). As expected, the staining of nuclear genomic DNA by PicoGreen was not changed.

To further investigate the presence of cytosolic RNA:DNA hybrids, we stained cells using the RNA:DNA hybrid-specific antibody S9.6 (25). In agreement with the PicoGreen results, S9.6 staining of tumor cells (A549, LoVo, HCT116, HT29, HeLa, and THP-1) and human fetal lung fibroblast cells (MRC-5) showed the presence of RNA:DNA hybrids in the cytosol and, to a lesser extent, in the nucleus (Fig. 1, *D* and *E*, and Fig. 2, *A* and *B*). As RNA:DNA hybrids can also form during replication of mitochondrial DNA (32), we co-stained cells with the mitochondria-specific vital dye MitoTracker. Three-dimensional surface rendering of confocal images showed that the majority of RNA:DNA hybrids were localized outside of mitochondria (Fig. 1*E*). Pretreatment of cells with RNase H prior to S9.6 staining significantly reduced the cytosolic RNA:DNA hybrid staining (Fig. 1, *D–F*). Antibody S9.6 only partially co-stained with PicoGreen (Fig. 1*G*), suggesting that PicoGreen stains cytosolic dsDNA and RNA:DNA hybrids in A549 cells. Consistent with this possibility, staining of A549 cells using a dsDNA-specific antibody showed the presence of cytosolic dsDNA, which partially co-localized with the cytosolic PicoGreen staining (Fig. 2*C*). In summary, our data show that RNA:DNA hybrids are constitutively present in the cytosol of all tested cells.

##### Presence of Cytosolic RNA:DNA Hybrids Depends on Pol III

**FIGURE 3.**
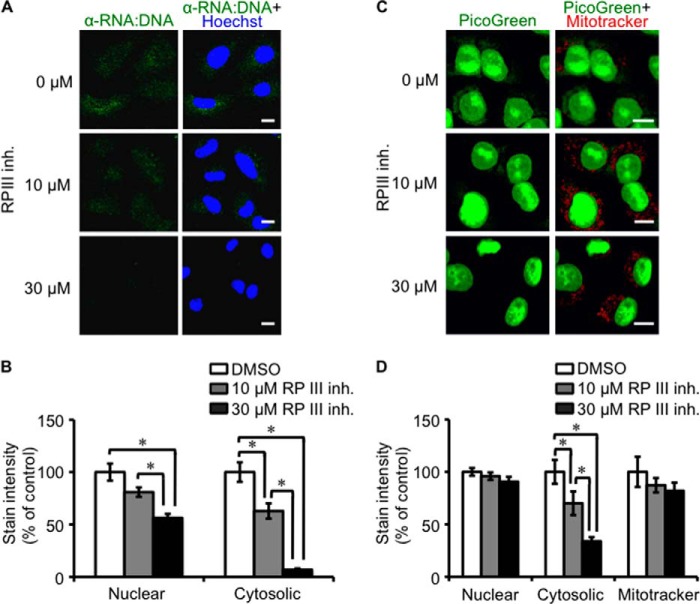
**Pol III is essential for the presence of cytosolic RNA:DNA hybrids.** *A*, A549 cells were treated with the indicated concentration of the Pol III inhibitor (*RP III inh.*) ML-60218 for 3 h before staining with 10 μl/ml PicoGreen (*green*) for 1 h and with 100 nm MitoTracker (*red*) for 30 min. *Scale bars* = 10 μm. *B*, cytosolic and nuclear intensity quantification of the images shown in *A*. A two-tailed Wilcoxon test was performed. *Error bars* represent S.E. *, *p* < 0.05. *C*, A549 cells were treated with the indicated concentrations of the Pol III inhibitor ML-60218 for 3 h before staining with RNA:DNA hybrid-specific S9.6 antibody (*green*) and Hoechst (*blue*). *D*, cytosolic and nuclear intensity quantification of the images shown in *C*. A two-tailed Wilcoxon test was performed.

RNA:DNA hybrids can occur during DNA transcription (33). In addition, cytosolic DNA is transcribed by Pol III in the cytosol and potentially in the nucleus into an RNA:DNA hybrid and dsRNA intermediate (22, 23). To understand the mechanism by which cytosolic RNA:DNA hybrids are generated and regulated, A549 cells were treated with Pol III inhibitors prior to staining with S9.6 or PicoGreen (34). Treatment of A549 cells with the Pol III inhibitor ML-60218 decreased the cytosolic RNA:DNA hybrid staining at doses above the published half-maximal inhibitory concentration (IC_50_), but had no effect on nuclear PicoGreen staining and affected RNA:DNA hybrid staining at doses above IC_50_ (Fig. 3). Treatment of A549 cells with α-amanitin, a Pol II inhibitor, was toxic to cells compared with the Pol III inhibitor ML-60218.

**FIGURE 4.**
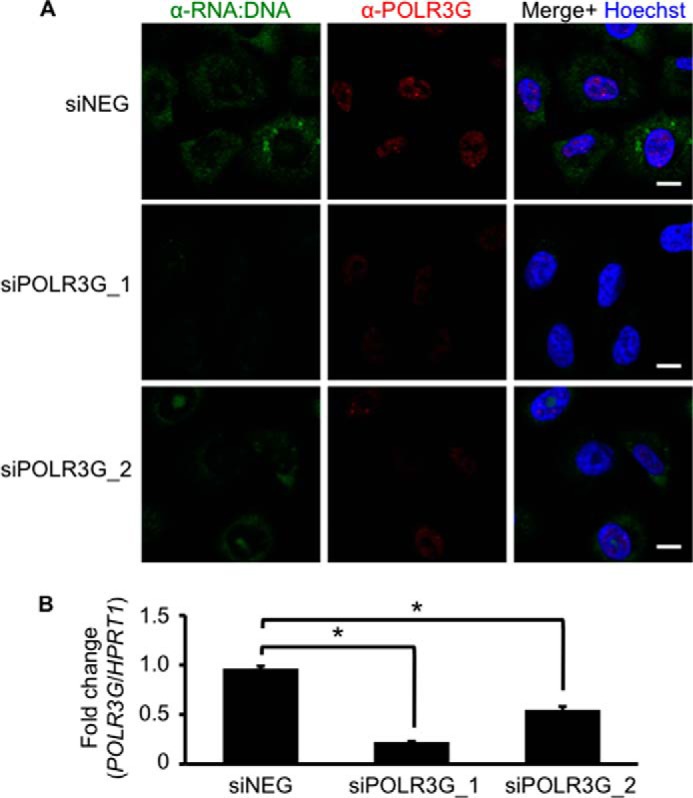
**Genetic knockdown of Pol III decreases cytosolic RNA:DNA hybrid levels.** *A*, A549 cells were transfected with 25 nm negative control siRNA (*siNEG*) or siRNA against POLR3G (siPOLR3G_1 and siPOLR3G_2). At 72 h after transfection, cells were stained for RNA:DNA hybrids (*green*) and POLR3G (*red*) in the presence of Hoechst (*blue*). *Scale bars* = 10 μm. *B*, some cells in *A* were subjected to RNA isolation for measurement of *POLR3G* mRNA expression with respect to *HPRT1*. Student's one-tailed *t* test was performed. *Error bars* represent S.E. *, *p* < 0.05.

To investigate if the genetic inhibition of Pol III also reduced RNA:DNA hybrid levels, A549 cells were transfected with siRNA against POLR3G (siPOLR3G_1 and siPOLR3G_2), a subunit of the Pol III complex, or negative control siRNA. Knockdown of POLR3G protein expression resulted in the disappearance of cytosolic RNA:DNA hybrids by immunofluorescence staining, consistent with reduced *POLR3G* mRNA gene expression (Fig. 4). In contrast, negative control siRNA did not affect cytosolic RNA:DNA hybrid levels or POLR3G expression. This corroborated with previous results of Pol III chemical inhibition, which decreased the presence of RNA:DNA hybrids.

**FIGURE 5.**
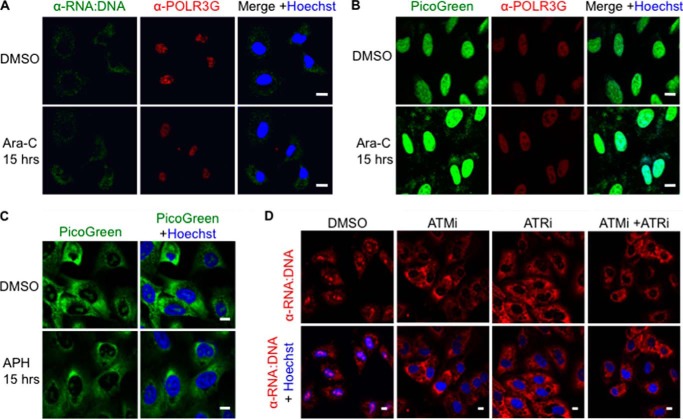
**Levels of cytosolic RNA:DNA hybrids are not modulated by genotoxic replication inhibitors.** *A*, A549 cells were treated with the genotoxic DNA replication inhibitor Ara-C at 10 μm for 15 h and stained for RNA:DNA hybrids (*green*) and POLR3G (*red*) in the presence of Hoechst (*blue*). *Scale bars* = 10 μm. *B*, A549 cells were treated with Ara-C as in *A* and stained with 10 μl/ml PicoGreen (*green*), POLR3G-specific antibody (*red*), and Hoechst (*blue*). *C*, A549 cells were treated with 4 μm aphidicolin (*APH*), an inhibitor of DNA polymerases, for 15 h and stained for RNA:DNA hybrids (*green*) in the presence of Hoechst (*blue*). *D*, A549 cells were treated with 10 μm ATM inhibitor (*ATMi*), ATR inhibitor (*ATRi*), or both for 15 h. Cells were stained for RNA:DNA hybrids (*red*) in the presence of Hoechst (*blue*).

In contrast to Pol II, which is localized exclusively in the nucleus, a fraction of Pol III is present in the cytosol. To investigate if cytosolic Pol III contributes to the generation of RNA:DNA hybrids in the cytosol, we co-stained A549 cells for POLR3G, a subunit of the Pol III complex, and RNA:DNA hybrids (35). No significant co-staining of POLR3G and S9.6 or PicoGreen was observed in the cytosol of A549 cells (Fig. 5*A*). Furthermore, POLR3G was localized largely in the nucleus, suggesting that the presence of cytosolic RNA:DNA hybrids depends on nuclear Pol III activity.

##### Presence of Cytosolic RNA:DNA Hybrids Is Independent of DNA Damage

RNA:DNA hybrids can cause stalling of the replication fork and formation of dsDNA breaks (1, 36, 37). To test if stalling of replication forks and the associated DNA damage contribute to the presence of RNA:DNA hybrids in the cytosol, A549 cells were treated with Ara-C, a genotoxic DNA replication inhibitor used to treat leukemia, and aphidicolin, an inhibitor of DNA polymerases (38). Treatment with Ara-C or aphidicolin had no effect on the level of cytosolic RNA:DNA hybrids in A549 cells (Fig. 5, *A* and *C*). Moreover, Ara-C treatment did not increase the co-localization of POLR3G and RNA:DNA hybrids (Fig. 5, *A* and *B*).

To test if the cellular response to DNA damage is required for the presence of cytosolic RNA:DNA hybrids, we inhibited ATM and ATR, two kinases that initiate the DDR. We previously found that the presence of dsDNA in the cytosol of B-cell lymphoma cells depends on the DDR (9). In contrast, inhibition of the DDR had no effect on the presence of RNA:DNA hybrids in the cytosol (Fig. 5*D*). Hence, unlike cytosolic dsDNA, RNA:DNA hybrid levels in the cytosol depend on Pol III, but not DNA damage or the DDR.

##### Cytosolic RNA:DNA Hybrids Bind Members of the miRNA Processing Machinery

**FIGURE 6.**
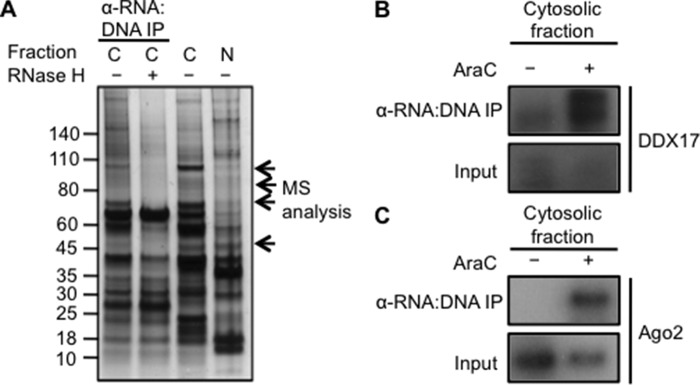
**Cytosolic RNA:DNA hybrids interact with miRNA machinery proteins.** *A*, cytosolic fractions of A549 cells were subjected to immunoprecipitation (*IP*) using RNA:DNA hybrid-specific antibody S9.6. Part of the cytosolic fraction was pretreated with 0.5 units/ml RNase H. Immunoprecipitated proteins were detected by SDS-PAGE and silver staining. The indicated bands were analyzed by mass spectrometry. *C*, cytosolic; *N*, nuclear. *B* and *C*, A549 cells were treated with DMSO or 10 μm Ara-C for 15 h and harvested for cell fractionation after fixation. Cytosolic fractions were subjected to immunoprecipitation with RNA:DNA hybrid-specific antibody S9.6. Immunoblot analysis was carried out on immunoprecipitated proteins probed with antibodies specific for DDX17 (*B*) and AGO2 (*C*).

To examine whether RNA:DNA hybrids interact with proteins in the cytosol, we performed immunoprecipitation experiments using the RNA:DNA-specific antibody S9.6 in cytosolic extracts of A549 cells. A fraction of the extracts were treated with RNase H before analysis to verify RNA:DNA hybrid-specific binding of proteins. Analysis by mass spectrometry identified DDX5/DDX17, AGO2, and BRCA1 (breast cancer susceptibility gene 1) as proteins that immunoprecipitated in an S9.6-dependent manner (Fig. 6*A*). All three proteins are part of the miRNA processing machinery (39). Immunoblot analyses of immunoprecipitated proteins showed that DDX17 binding was consistent with the mass spectrometry analysis (Fig. 6*B*). AGO2 is part of the miRNA-mediated DDR (40) and increases interaction with repair molecules during double-strand break repair (41). Ara-C treatment increased the interaction of S9.6 with AGO2 proteins (Fig. 6*C*). Consequently, components of the miRNA processing machinery were found to interact with cytosolic RNA:DNA hybrids in the absence of DNA damage and increased interaction in the presence of DNA damage.

##### Pol III Regulates the Expression of Specific miRNAs

**FIGURE 7.**
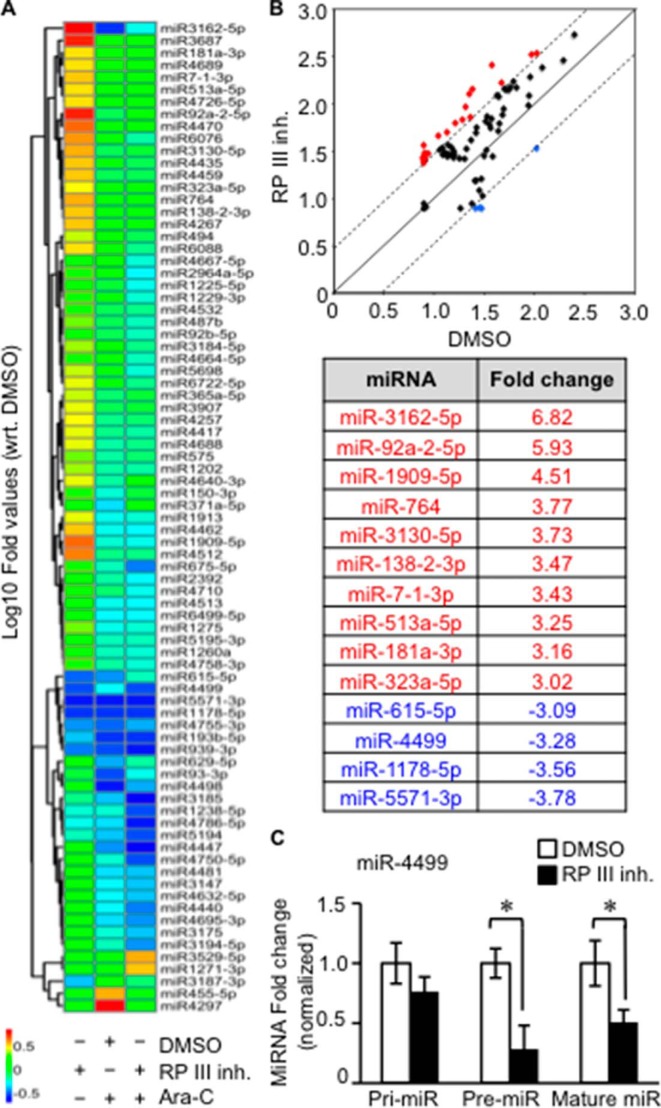
**Deregulation of miRNAs by Pol III inhibition.** *A*, A549 cells were treated with the Pol III inhibitor (*RP III inh.*) ML-60218 at 10 μm or DMSO for 24 h. Some cells were further treated with Ara-C for 15 h. A heat map of the global miRNA expression profile from total RNA extracted after treatment is shown. All expression profiles were quantile-normalized, and then -fold values with reference to the control (DMSO) were used for plotting this heat map. 81 probes had at least 3-fold differential expression in any pair of conditions and were preselected for this plot. *wrt* indicates normalized to. *B*, scatterplot of miRNA expression profiles between control (DMSO) and ML-6028-treated samples. The decision surface was plotted for at least a 3-fold change to or from the control. Highly up-regulated and down-regulated miRNAs after Pol III inhibitor treatment are identified in the *table* (*lower rows*). *C*, A549 cells were treated with 10 μm ML-60218 or DMSO for 24 h. The expression levels of primary (*Pri-miR*), precursor (*Pre-miR*), and mature miR-4499 from isolated RNA were examined. Student's two-tailed *t* test was performed. *Error bars* represent S.E. *, *p* < 0.05.

We next sought to gain insights into the Pol III-dependent mechanisms leading to the presence of cytosolic RNA:DNA hybrids. Our data support the possibility that Pol III-mediated transcription of miRNAs is associated with the presence of cytosolic RNA:DNA hybrids. Pol III is able to transcribe a subset of miRNAs in a cell type-specific manner (42) or interact with canonical genes within a chromosome location that also encodes miRNAs (43). To determine whether a subset of miRNAs are dependent on Pol III, we examined the Pol III-dependent miRNA expression profile in A549 cells by comprehensive miRNA array analysis. To distinguish Pol III effects from RNA:DNA hybrid-induced DNA damage, cells were treated with the genotoxic DNA replication inhibitor Ara-C or the Pol III inhibitor ML-60218. A total of 81 differentially expressed miRNAs were identified after comparison across the treatment groups (Fig. 7*A*). Strikingly, treatment of cells with the Pol III inhibitor resulted in significant down-regulation of only four miRNAs: miR-615-5p, miR-1178-5p, miR-4499, and miR-5571-3p (Fig. 7, *A* and *B*). The expression of miR-615-5p, miR-1178-5p, and miR-5571-3p was also decreased after Ara-C treatment, suggesting that the expression of these miRNAs is also down-regulated by DNA damage. In contrast, miR-4499 is likely to be transcribed directly by Pol III, as Ara-C had no effect on its expression. Surprisingly, the expression of 10 miRNAs was up-regulated after treatment with the Pol III inhibitor ML-60218, but not Ara-C (Fig. 7, *A* and *B*). To confirm that Pol III directly regulates miR-4499 expression, miRNA expression of miR-4499 was measured after Pol III treatment. Mature miR-4499 expression decreased after treatment, corresponding to miRNA microarray data (Fig. 7*C*). Precursor miR-4499 also decreased significantly, whereas primary miR-4499 was not significantly affected (Fig. 7*C*). This suggests that Pol III does not directly transcribe primary miR-4499, but might regulate Drosha processing of precursor miR-4499 to affect mature miR-4499 expression. In summary, our data indicate that Pol III regulates the expression of a limited number of miRNAs, which may contribute to the presence of cytosolic RNA:DNA hybrids in A549 cells.

**FIGURE 8.**
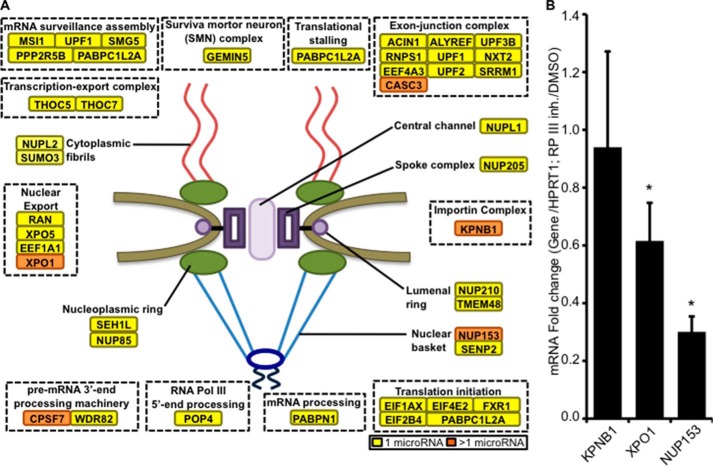
**RNA transport and mRNA surveillance pathways are potential targets of Pol III-modulated miRNAs.** *A*, a list of predicted miRNAs regulated by Pol III were analyzed for potential gene targets by using the online resource DIANA-mirPath. *Yellow boxes* indicate genes that are regulated by one miRNA in response to Pol III inhibition. *Orange boxes* indicate potential target genes regulated by more than one miRNA upon Pol III inhibition. *B*, mRNA expression levels of three potential genes, each targeted by more than one miRNA. Total RNA was extracted from A549 cells after treatment with the Pol III inhibitor (*RP III inh.*) ML-60218 at 10 μm or DMSO for 24 h and subjected to real-time PCR analysis. Normalization was done with respect to the *HPRT1* housekeeping gene and further compared with DMSO-treated cells. A one-tailed Wilcoxon test was performed. *Error bars* represent S.E. *, *p* < 0.05.

To investigate the potential transcripts targeted by Pol III-modulated miRNAs, a miRNA target prediction and pathway analysis was performed using the DIANA-mirPath software (27). Among the top ranked pathways, the RNA transport and RNA surveillance pathways were identified to contain predicted genes that are targeted by two or more ML-60218-induced miRNAs (Fig. 8*A*, *orange boxes*), whereas other transcripts are potentially targeted by a single miRNA (*yellow boxes*). Hence, RNA transport or stability may contribute to the presence of RNA:DNA hybrids. To test the predictability of the DIANA-mirPath software, three genes (*KPNB1*, *XPO1*, and *NUP153*) were selected for mRNA expression detection after Pol III inhibition. The gene expression of *XPO1* and *NUP153* was found to be down-regulated by 1.6- and 3.3-fold, respectively (Fig. 8*B*).

##### Exportin-1 Regulates Transport of RNA:DNA Hybrids from the Nucleus to the Cytosol

**FIGURE 9.**
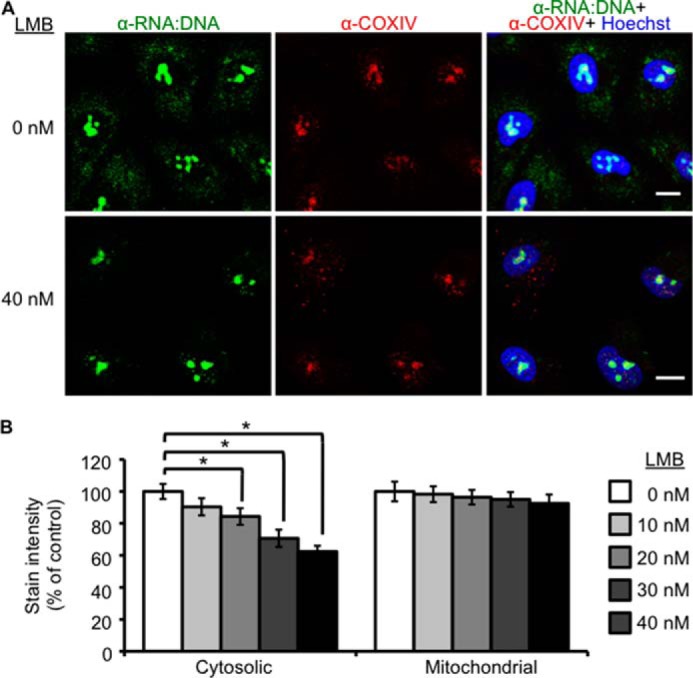
**Exportin-1 function is required for the presence of cytosolic RNA:DNA hybrids.** *A*, A549 cells were treated with the indicated concentrations of the exportin-1 inhibitor leptomycin B (*LMB*) for 16 h before staining with RNA:DNA hybrid-specific antibody S9.6 (*green*), COX IV (*red*), and Hoechst (*blue*). *Scale bars* = 10 μm. *B*, A549 cells were treated with increasing concentrations of leptomycin B (0, 10, 20, 30, and 40 nm) for 16 h before staining with RNA:DNA hybrid-specific antibody S9.6, COX IV, and Hoechst. Microscope images were quantified for cytosolic and mitochondrial intensity of cells. Student's two-tailed *t* test was performed. *Error bars* represent S.E. *, *p* < 0.05.

Of the identified genes that were down-regulated in response to Pol III inhibition, *XPO1* encodes for exportin-1, a protein involved in RNA transport and miRNA processing (44, 45). To test if exportin-1 is involved in transport of nuclear RNA:DNA hybrids to the cytosol, cells were treated with leptomycin B, an XPO1 inhibitor. Increasing concentrations of leptomycin B decreased accumulation of cytosolic RNA:DNA hybrids, whereas cytosolic COX IV remained unchanged (Fig. 9), suggesting that XPO1 function is required for the presence of RNA:DNA hybrids.

## DISCUSSION

Here, we have shown the existence of endogenous RNA:DNA hybrids in the cytosol of a variety of human cells, including cancer cells. We previously found that the presence of ssDNA and dsDNA in the cytosol of B-cell lymphomas depends on DNA damage and the ensuing DDR (9). In contrast, treatment of cells with genotoxic agents or blocking of the DDR had no effect on the levels of cytosolic RNA:DNA hybrids, suggesting that the presence of RNA:DNA hybrids is regulated by different pathways. Consistent with this conclusion, inhibition of Pol III led to the disappearance of RNA:DNA hybrids in the cytosol. Interestingly, Pol III inhibitors also abrogated cytosolic PicoGreen staining, which stained cytosolic dsDNA in A549 cells, suggesting that Pol III contributes to the presence of dsDNA in A549 cells. It is possible that Pol III-dependent R-loops, which contain RNA:DNA hybrids, contribute to the presence of cytosolic dsDNA in tumor cells by stalling replication forks, resulting in DNA damage and activation of the DDR (37). In agreement with this possibility, it was recently shown that the DDR is activated in cells that are deficient in the R-loop-resolving enzyme RNase H2 (46). Furthermore, overexpression of *Rnaseh1*, which degrades the RNA strand in RNA:DNA hybrids, decreases the levels of cytosolic ssDNA and dsDNA in tumor cells.^4^

In previously published studies, virus- or bacteria-derived cytosolic RNA:DNA hybrids were described in human cells (12, 13). Our data show that RNA:DNA hybrids can also exist in the cytosol of non-infected cells. All the cells used in our study were cultured under sterile conditions and were mycoplasma-free. Cells were further treated with Plasmocin to exclude potential undetected mycoplasma contaminations, suggesting that cytosolic RNA:DNA hybrids are not a result of infection of the tested cells. RNA:DNA hybrids were also reported to form in the mitochondrial genome of non-infected cells (47, 48). Co-staining of cells with mitochondria-specific dyes or markers showed that extramitochondrial RNA:DNA hybrids are present in human cells. Furthermore, inhibition of Pol III, which has not been found to localize to mitochondria or extranuclear RNA:DNA hybrids, abrogated the presence of RNA:DNA hybrids. In addition, exportin-1 inhibition of nuclear export decreased cytosolic RNA:DNA hybrids, suggesting that RNA:DNA hybrids are derived from the nucleus. In summary, our data suggest that nuclear Pol III activity is required for the presence of RNA:DNA hybrids in the cytosol of non-infected cells.

Each polymerase in the RNA polymerase family of proteins has defined transcriptional roles (49). Pol I transcribes ribosomal RNA; Pol II transcribes protein-encoding mRNA; and Pol III transcribes 5 S rRNA, tRNA, and certain retroelements. Noncoding RNAs can be transcribed by all three polymerases in eukaryotic cells (50). Strikingly, the inhibition of Pol III, but not DNA polymerases, abrogated the presence of cytosolic RNA:DNA hybrids, suggesting that Pol III transcripts are required for the existence of such structures in the cytosol. In contrast, the level of nuclear RNA:DNA hybrids was not decreased in response to Pol III inhibition, consistent with previous reports that the majority of RNA:DNA hybrids in the nucleus are transcribed by Pol II (51).

Immunoprecipitation experiments suggest that cytosolic RNA:DNA hybrids bind to the DDX17-containing Drosha miRNA complex (52). It is therefore conceivable that cytosolic RNA:DNA hybrids consist of Pol III-transcribed miRNAs. Analysis of the miRNA expression profile showed that Pol III modulated the expression of a small number of miRNAs, most of which were up-regulated upon Pol III inhibition, suggesting a complex regulation of these miRNAs by Pol III in A549 cells. Surprisingly, Pol III inhibition reduced the expression of only four miRNAs. Three of the four miRNAs were also down-regulated by Ara-C, which does not modulate the levels of cytosolic RNA:DNA hybrids. Hence, our data suggest that cytosolic RNA:DNA hybrids would consist of a very limited number of Pol III-transcribed miRNAs, possibly only miR-4499. However, miR-4499 expression after Pol III treatment suggests that Pol III does not directly transcribe primary miR-4499, and Pol III may instead affect Dicer processing of precursor miR-4499. Alternatively, it is conceivable that Pol III-modulated miRNAs target transcripts of genes, which regulate the presence of cytosolic RNA:DNA hybrids. Consistent with this possibility, many Pol III-modulated miRNAs potentially target RNA transport and mRNA stability pathways. Of the affected target genes, exportin-1 inhibition was found to prevent accumulation of cytosolic RNA:DNA hybrids, suggesting that Pol III may have multiple functions in the generation of RNA:DNA hybrids and regulation of miRNAs that modulate the transport of nuclear RNA:DNA hybrids into the cytosol. Cytosolic RNA:DNA hybrids may also be generated by the transcription of cytosolic DNA by Pol III, which was reported to detect cytosolic DNA and induce type I interferons through the RIG-1 pathway (20–22). However, Pol III did not co-localize with cytosolic DNA or cytosolic RNA:DNA hybrids. Furthermore, inhibition of Pol III did not reduce type I interferon transcript levels (data not shown), consistent with the conclusion that neither Pol III nor other type I interferon-inducing sensors recognize DNA in the cytosol of A549 cells. In agreement with this observation, no known cytosolic DNA or RNA sensor precipitated with RNA:DNA hybrids. Finally, it is conceivable that reverse transcription of Pol III-transcribed retroelements produces RNA:DNA intermediates in the cytosol, similar to replicating RNA viruses (4, 6, 53).

In summary, we have shown that Pol III activity leads to the presence of RNA:DNA hybrids in human cells. The functional role of RNA:DNA hybrids remains to be further investigated, but RNA:DNA hybrids may modulate cellular functions or contribute to immune recognition by activating TLR9 or the NLRP3 inflammasome in dendritic cells (12, 13).

## Footnotes

4 Y. J. Shen, N. Le Bert, A. A. Chitre, C. X. Koo, X. H. Nga, S. S. W. Ho, K. J. Ishii, D. H. Raulet, and S. Gasser, submitted for publication.

## Footnotes

3 The abbreviations used are:

DDR: DNA damage response
Pol III: RNA polymerase III
miRNA/miR: microRNA
DMSO: dimethyl sulfoxide
COX IV: cytochrome *c* oxidase
Bis-Tris: 2-[bis(2-hydroxyethyl)amino]-2-(hydroxymethyl)propane-1,3-diol.
